# Area and distance from mainland affect in different ways richness and phylogenetic diversity of snakes in Atlantic Forest coastal islands

**DOI:** 10.1002/ece3.5019

**Published:** 2019-03-14

**Authors:** José Thales da Motta Portillo, Lilian Sayuri Ouchi‐Melo, Lucas Batista Crivellari, Thiago Alves Lopes de Oliveira, Ricardo J. Sawaya, Leandro da Silva Duarte

**Affiliations:** ^1^ Instituto de Biociências, Letras e Ciências Exatas Universidade Estadual Paulista “Júlio de Mesquita Filho” São José do Rio Preto Brazil; ^2^ Department of Biology, City College of New York City University of New York New York New York; ^3^ Departamento de Zoologia Universidade Federal do Paraná Curitiba Brazil; ^4^ Depto de Ecologia, Laboratório de Ecologia Filogenética e Funcional Universidade Federal do Rio Grande do Sul Porto Alegre Brazil; ^5^ Centro de Ciências Naturais e Humanas Universidade Federal do ABC São Bernardo do Campo Brazil

**Keywords:** Atlantic forest, distance, Island Biogeography, phylogenetic composition, snakes, species‐area

## Abstract

**Aim:**

The Theory of Island Biogeography posits that ecological and evolutionary processes regulate species richness of isolated areas. We assessed the influences of an island area and distance from the mainland on species richness, phylogenetic diversity, and phylogenetic composition of snakes on coastal islands.

**Location:**

Coastal islands of the megadiverse Atlantic Forest in southeastern Brazil.

**Methods:**

We compiled the species composition of 17 coastal islands in southeastern Brazil. Species richness and phylogenetic diversity were calculated for each island. Phylogenetic composition was measured using principal coordinates of phylogenetic structure. We then employed generalized linear models to test the influence of area and distance from the mainland on the diversity metrics.

**Results:**

We found a prominent influence of area on species richness, whereas phylogenetic diversity was more affected by distance from the mainland. Snake clades were distinctly associated with area and distance. The Boidae family was associated with nearer and larger islands, whereas Elapidae was broadly distributed. Distance from the mainland was associated with the distribution of Dipsadidae, whereas Colubridae was influenced by both the area and distance. The Viperidae family attained higher values of phylogenetic diversity in smaller and more remote islands.

**Main conclusions:**

This island system conserved a considerable piece of snake richness from southeastern Brazil, including island endemic species. Area and distance from the mainland were important drivers of snake diversity in the Atlantic Forest coastal islands. However, these predictors affected the different components of diversity in different ways. Phylogenetic composition analysis enables us to understand how basal nodes contributed to high levels of phylogenetic diversity on smaller and farther islands regardless of the decrease in species richness.

## 1 INTRODUCTION

MacArthur and Wilson's Theory of Island Biogeography proposes that ecological and evolutionary processes, such as colonization, speciation, and stochastic extinction, regulate species richness in isolated areas by creating an equilibrium between the gain and/or exclusion of species (MacArthur & Wilson, 1963, 1967). The fundamental prediction of the Island Biogeography theory is that the rates of processes involved are dependent on the geographical context, whereas island area and isolation play significant roles in the species richness equilibrium (Patino et al., 2017). Thus, species richness is expected to decrease in smaller islands farther from the mainland due to greater local extinctions and less immigration, and to increase in larger islands closer to the mainland because of the high levels of immigration and larger area available for foraging (MacArthur & Wilson, 1963, 1967; Warren et al., 2015). Larger islands also tend to hold larger populations by reducing the probability of stochastic extinctions (Whittaker & Fernández‐Palacios, 2007).

Most studies testing predictions of the Theory of Island Biogeography have focused on species richness patterns (Kadmon & Pulliam, 1993; Kalmar & Currie, 2006; Lindgren & Cousins, 2017). As an example, Centeno, Sawaya, and Marques (2008) corroborated the Theory of Island Biogeography by comparing the structures (species richness, composition, and dominance) of snake assemblages in a Brazilian tropical island system, which suggested that relictual snake populations from the continental lowland and Serra do Mar coastal range were part of the island's composition. However, diversity on islands could also be related to evolutionary processes, such as the time available for speciation and rates of extinction in the regional species *pool* (Losos & Schluter, 2000; Rabosky & Glor, 2010). Furthermore, strong evidence exists of fast speciation within islands (Amaral, 1921; Barbo et al., 2016; Barbo, Grazziotin, Sazima, Martins, & Sawaya, 2012), which contributes to the assembling process in the area, with endemic species being generated in some island systems.

Lomolino (2000) notes some paradigms and limitations of Island Biogeography Theory, including spatial and temporal scales, immigration filters (e.g., intervening landscapes or seascapes and environmental conditions regarding island size), the neutral theory, and the challenge of evolutionary approaches to better clarify the assembly process in insular community structures. However, even the combination of molecular phylogenies and species composition has not been well explored to investigate the role of speciation in driving island community structures.

We have experienced an era of rapidly emerging community phylogenetic tools, making it feasible to test island biogeography predictions through an evolutionary timescale. Recently, Pyron and Burbrink (2014) employed community phylogenetic tools to analyze patterns of snake diversity of 510 islands around the globe and demonstrated that colonization was the main process explaining most of species richness distribution patterns in islands. Furthermore, they verified in situ diversification as rare and not contributing to island species richness. These authors also have shown that phylogenetic diversity on islands is associated with isolation and climate but not area. Herein, we advance our understanding by identifying the influence of area and distance from the mainland on snake lineages in a megadiverse tropical biodiversity hotspot. We also shed light on the possible processes responsible for island community assembly under a phylogenetic approach.

We aimed to assess the accuracy of the predictions of the Theory of Island Biogeography for the determination of species richness, phylogenetic diversity, and lineage composition of snakes among the coastal islands in the Atlantic Forest hotspot. We sought to answer the following questions: (a) Do species richness and phylogenetic diversity of coastal islands increase in larger and closer islands? and (b) How different are the phylogenetic components regarding the variation in island area and distance from the mainland? We expected that species richness and phylogenetic diversity would present a positive relationship to area and a negative association to distance from the mainland, as predicted by Island Biogeography Theory, but different lineages should affect richness and phylogenetic diversity of snakes in islands differently.

## 2 MATERIALS AND METHODS

### 2.1 Study area and database

We analyzed 17 coastal islands located in the Atlantic Forest domain in São Paulo state, southeastern Brazil (23°23'00'' to 25°19'13''S and 44°43'44'' to 48°06'00''W). Precipitation in the islands ranges from 90 to 330 mm/year, and the average temperatures ranges from 18 to 27°C (Cicchi, Sena, Peccinini‐Seale, & Duarte, 2007). The climate is considered tropical by Peel, Finlayson, and McMahon (2007). These islands conserve dense ombrophilous forest and herbaceous shrub phytophysiognomies, as well as “restinga” vegetation (Cicchi, Serafim, Sena, Centeno, & Jim, 2009; Kurtz et al., 2017; Rocha, Bergallo, Conde, Bittencourt, & Santos, 2008).

The number of species was recorded from Cicchi et al. (2007), Centeno et al. (2008), Rocha et al. (2008), Cicchi et al. (2009), and Barbo et al. (2012). We considered, as a regional pool, 108 species with potential occurrence in the dense ombrophilous forests of the region (see Zaher et al., 2011), which makes them potential colonizers for studied islands. To characterize island areas and distance from the mainland, we extracted data from Cicchi et al. (2007) (Table 1). We considered, as the local pool, the species composition of each island. In this way, we provided a complete list of the 40 species included in our analyses (Table 1 and Supporting information Appendix S1).

**Table 1:** Coastal islands of the Atlantic Forest in southeastern Brazil and dataset of metrics used in this study

Islands	Area	Distance	Richness	PSV	PCPS 1	PCPS 3	PCPS 4
1‐Alcatrazes	135	33.4	4	0.64	0.008	0.07	0.04
2‐Anchieta	828	0.49	6	0.65	−0.13	−0.005	−0.05
3‐Barnabé	173.4	0.01	2	0.59	0.2	0.06	−0.09
4‐Bom Abrigo	154	3.55	2	0.81	−0.18	0.14	0.07
5‐Búzios	755	24.09	4	0.68	−0.04	−0.02	−0.02
6‐Cananeia	13.7	0.24	16	0.48	0.16	−0.04	0.002
7‐Cardoso	22,500	0.08	25	0.42	0.2	−0.02	−0.03
8‐Comprida	20,000	0.31	12	0.53	0.09	−0.07	−0.002
9‐Couves	64.5	2.53	1	0.0	0.18	0.15	−0.15
10‐Mar Virado	119	2	3	0.57	−0.18	−0.09	−0.15
11‐Porchat	15	0.23	10	0.55	0.01	−0.07	−0.03
12‐Porcos	24.2	0.74	1	0.0	−0.39	−0.21	−0.008
13‐Queimada Grande	430	34.8	2	0.81	−0.21	0.15	0.07
14‐Santo Amaro	14,000	0.05	21	0.50	0.16	−0.07	0.17
15‐São Sebastião	33,600	1.76	22	0.49	0.17	−0.06	0.16
16‐São Vicente	6,000	0.12	22	0.44	0.19	−0.04	−0.02
17‐Vitória	221.3	37.97	4	0.69	−0.24	0.13	0.05

Note

Predictor variables: area (hectares) and distance from mainland (kilometers) (from Cicchi et al., 2007); snake richness and phylogenetic diversity (*Phylogenetic* *Species Variability*—PSV); and assessed principal coordinates of phylogenetic structure (PCPS).

### 2.2 Phylogenetic diversity and composition

To estimate phylogenetic diversity, we used a consensus phylogenetic tree from Tonini, Beard, Ferreira, Jetz, and Pyron (2016) encompassing 9,755 species of Squamate reptiles. Missing species (*Echinanthera bilineata*, *Thamnodynastes nattereri,* and *Xenodon merremii*) in this phylogeny were conservatively placed in polytomies within genera, along with their sister species, by using the package phytools (Revell, 2018) of R software version 3.2.1. *Bothrops otavioi* was manually moved in Mesquite Software (Maddison & Maddison, 2011), with the related island endemic species from the “jararaca” group (see Barbo et al., 2012) (Figure 3).

We estimated phylogenetic diversity by using the richness‐independent metric *Phylogenetic* *Species Variability* (PSV) (Helmus, Bland, Williams, & Ives, 2007). PSV quantifies the decrease in phylogenetic relatedness according to similarities shared by all species in a community (herein in each island), regardless of the total number of species. Briefly, PSV is an index based on the phylogenetic covariance expected for the related taxa, which is scaled between 0, where all species are closely related, and 1, where all species present a similar trend in the degree of relatedness, as with a star phylogeny (Helmus & Ives, 2012). We used the package picante (Kembel et al., [Link]) to calculate the PSV index.

To evaluate the variation in lineage composition among islands, we used the principal coordinates of the phylogenetic structure analysis (PCPS; see Duarte, 2011) calculated in the PCPS package (Debastiani, 2015; Debastiani & Duarte, 2014). This approach allows verification of the main orthogonal gradient of the variation in the phylogenetic structure among the islands. The phylogenetic composition matrix was calculated using phylogenetic fuzzy weighting (see Pillar & Duarte, 2010; Duarte, Debastiani, Freitas, & Pillar, 2016) converted into a Bray Curtis dissimilarity matrix. The next step was to apply a principal coordinate analysis (PcoA) to generate principal coordinates of phylogenetic structure (PCPS) for each island. Each PCPS is a vector describing an orthogonal phylogenetic gradient of the lineages included (Duarte, 2011; Duarte, Prieto, & Pillar, 2012). PCPS with higher eigenvalues depicts the monotonic gradient regarding basal nodes of the phylogenetic tree (Duarte et al., 2012). As the PCPS eigenvalues decrease, finer phylogenetic gradients concerning more terminal nodes are described (Duarte et al., 2012). Thus, to represent the phylogenetic composition, we selected the first two PCPS vectors with a significant association with predictor variables that represent the greater variation on phylogenetic composition structure regarding area and distance from the mainland.

### 2.3 Data analysis

We evaluated the collinearity among predictor variables with variance inflation factor analyses (VIF; Zuur, Ieno, & Elphick, 2010), considering VIF < 3.0 as the threshold to exclude autocorrelated environmental predictors. After the VIF procedure, we standardized the predictors by scaling them to have the same range of variation (mean 0 and unit variance) to avoid potential type I and II errors.

To test the influence of island area and distance from the mainland on richness, phylogenetic diversity (PSV), and PCPS vectors, we used a generalized linear model (GLM) based on Akaike information criteria (AIC; Burnham & Anderson, 2002). The GLM model is a useful tool when the data exhibit nonconstant variance distribution or when no normal distribution of errors is present (Crawley, 2007). GLM is able to define the type of error distribution by applying the best model to improve the correlation between the predictors and response variables, which is the Gaussian distribution in this case. We applied two different types of null models to access the significance of the GLMs: in the first one, the site positions were randomly shuffled across the environmental gradient; and, in the second, the species were randomly shuffled among the phylogeny tips, generating a set of 999 null PCPS (Debastiani & Duarte, 2014). All analyses were performed in R software 3.2.1 (R Development Core Team, 2012).

We also tested spatial autocorrelation by Moran's I correlograms (Legendre & Legendre, 2012) for species richness, phylogenetic diversity (PSV index), and phylogenetic composition regarding PCPS vectors. Briefly, Moran's I is an index of similarity between values (herein all response variables) of two points, and such values of spatial autocorrelation are plotted as a function of distance classes on the abscissa (Legendre & Legendre, 2012). We chose a priori seven distance classes with equal number of sample units allocated in each class, which increases predictive power of the analysis (Legendre & Legendre, 2012). The significance test was based in 999 randomizations. We implemented Moran's I correlograms in the software *Spatial Analysis in Macroecology *(SAM; Rangel, Diniz‐Filho, & Bini, 2006; Rangel, Diniz‐Filho, & Bini, 2010).

## 3 RESULTS

We recorded 40 snake species belonging to five families on the 17 islands analyzed (see Supporting Information Appendix S1). The species pool of these islands represents approximately 37% of the regional pool (Zaher et al., 2011). The richest families were Dipsadidae and Colubridae, with 26 and seven species, respectively. “Ilha do Cardoso” exhibited the highest species richness (25 species; Table 1), whereas “Ilhas dos Porcos” and “Ilha das Couves” showed the lowest species richness (only one species) and phylogenetic diversity (Table 1). The highest phylogenetic diversity was recorded in “Bom Abrigo” and “Queimada Grande” islands (both with PSV = 0.81) (Table 1).

**Figure 1:**
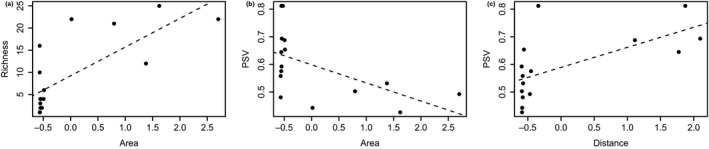
Relationships between island area (a and b) and distance from the mainland (c) on response species richness and phylogenetic diversity (*Phylogenetic* *Species Variability—*PSV; respectively). Species richness shows a significant positive association to area (*R* ^2^ = 0.53, *p* = 0.0005). Phylogenetic diversity presents a negative relationship with area (*R* ^2^ = 0.26, *p* = 0.03) and a positive relationship with distance from mainland (*R* ^2^ = 0.34, *p* = 0.01). See more details in Table 2

We found that island area per se explained 53% of species richness variation (*R* ^2^ = 0.53, *p* < 0.05, Figure 1a, Table 2). Our model was not improved by adding distance from the mainland, as area and distance together explained the same 53% of species richness variation (*p* = 0.001, Table 2). Distance itself was not important in explaining species richness variation (*p* = 0.10). Area had a greater importance in driving species richness variation pattern in all evaluated models (wAIC = 0.74, Table 2). Regarding phylogenetic diversity, area explained 26% (*p* = 0.03), and the distance from the mainland explained 34% of the PSV variation (*p* = 0.01, Figure 1b, Table 2). When considered together in the model, the area and distance explained 44% of the total PSV variation (*p* = 0.01, Table 2). Area and distance together presented the highest AIC weight (wAIC = 0.43, Table 2), but only distance from the mainland exhibited a great part of PSV explanation (wAIC = 0.40, Table 2).

**Table 2:** Influence of area and distance from the mainland on richness and phylogenetic diversity (PSV) of snakes recorded in the coastal islands of the Atlantic Forest in southeastern Brazil

Model	AIC	∆AIC	wAIC	*p*	*R* ^2^
Richness ~ Area^a^	114.3	0.0	0.74	0.0005	0.53
Richness ~ Dist	125.3	11	0.003	0.10	0.1
Richness ~ Area + Dist^a^	116.4	2.1	0.26	0.001	0.53
PSV ~ Area^a^	−19.5	1.9	0.17	0.03	0.26
PSV ~ Dist^a^	−21.3	0.2	0.40	0.01	0.34
PSV ~ Area + Dist^a^	−21.4	0.0	0.43	0.01	0.44

Notes

Likelihood measures with Gaussian distribution.

AIC: Akaike information criterion; ΔAIC: Difference of Akaike information criterion to each model from the most parsimonious model; wAIC: AIC weight for each model; *p*: probability; and *R* ^2^: adjusted coefficient of determination; Area: Island area in hectares; Dist: island distance from mainland in kilometers; Richness: number of species; and PSV: richness‐independent phylogenetic diversity (see Materials and Methods for details).

a Significant relationships of metrics and correspondent predictors.

The first four principal coordinates of phylogenetic structure (PCPS) accounted for 59%, 27%, 17%, and 13% of the total phylogenetic composition variation, respectively. The PCPS 1 had a significant relationship to area (*p*_site shuffle_ = 0.05, Table 3). We did not find any relationship between PCPS 2 and the predictors. PCPS 3 was associated with distance from the mainland (*p*_site shuffle_ = 0.02, Table 3), whereas PCPS 4 was significantly associated with the island area (*p*_site shuffle_ = 0.05; Table 3), as well as with the distance and area together (*p*_site shuffle_ = 0.04; Table 3).

**Table 3:** Environmental influence on phylogenetic composition of snakes (PCPS 1, PCPS 3 and PCPS 4) recorded in coastal islands of the Atlantic Forest in southeastern Brazil

Model	f.obs	*P*_site shuffle_	*P*_taxa shuffle_	AIC	∆AIC	wAIC	*P*	*R* ^2^
PCPS 1 ~ Area^a^	4.12	0.05	0.11	−5.7	0.0	0.52	0.05	0.18
PCPS 1 ~ Dist	2.52	0.13	0.3	−4.3	1.4	0.26	0.09	0.11
PCPS 1 ~ Area+Dist	2.68	0.09	0.21	−4.0	1.7	0.22	0.07	0.21
PCPS 3 ~ Area	2.25	0.15	0.36	−23.2	4.3	0.08	0.22	0.04
PCPS 3 ~ Dist^a^	7.5	0.02	0.05	−27.5	0.0	0.74	0.02	0.25
PCPS 3 ~ Area + Dist	4.11	0.04	0.12	−24.6	2.9	0.17	0.06	0.22
PCPS 4 ~ Area^a^	4.05	0.05	0.07	−30.7	0.6	0.4	0.05	0.16
PCPS 4 ~ Dist	1.08	0.34	0.4	−27.6	3.7	0.08	0.34	−0.002
PCPS 4 ~ Area + Dist^a^	4.5	0.04	0.05	−31.3	0.0	0.53	0.03	0.3

Notes

Likelihood measures with Gaussian distribution.

AIC: Akaike information criterion; ΔAIC: Difference of Akaike information criterion from most parsimonious model; wAIC: AIC weight for each model; *p*: probability; and *R* ^2^: adjusted coefficient of determination; Area: Island area in hectares; Dist: island distance from mainland (meters).

a Significant relationships of PCPS and correspondent predictors.

**Figure 2:**
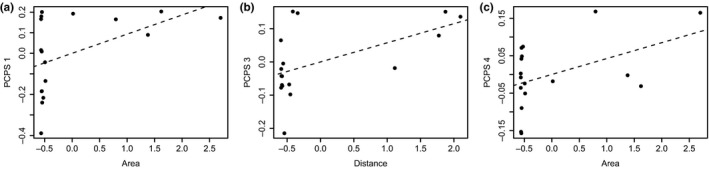
Relationships between principal coordinates of phylogenetic structure (PCPS) axes and predictor variables. (a) PCPS 1 and area (*R* ^2^ = 0.18, *p* = 0.05); (b) PCPS 3 and distance from mainland (*R* ^2^ = 0.25, *p* = 0.02); (c) PCPS 4 and area (*R* ^2^ = 0.16, *p* = 0.05). See more details in the Table 3

Island area explained 18% of the variation in the phylogenetic composition (*p* = 0.05, Figure 2a, Table 3) regarding PCPS 1 (basal nodes). On the other hand, distance from the mainland explained 25% of the variation in phylogenetic composition regarding PCPS 3 (*p* = 0.02, Figure 2b, Table 3). Area and distance together explained 30% of the variation in phylogenetic composition among the islands (*p* = 0.03; Table 3) regarding PCPS 4. The area explained 16% of the phylogenetic composition variation regarding PCPS 4 (*p* = 0.05; Figure 2c, Table 3).

**Figure 3:**
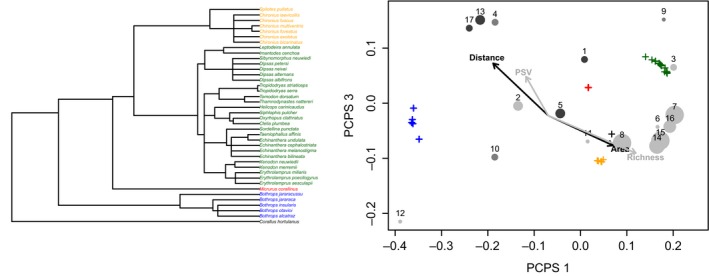
Ordination of coastal island assemblages of Atlantic Forest in southeastern Brazil (circles), and snake families (crosses) along axis 1 and 3 of a principal coordinate analysis of phylogenetic structure (PCPS; see details in text). Numbers correspond to the islands listed in Table 1, and point sizes correspond to the total area of each island. Gray shades are related to distance from the mainland, with darker shades corresponding to greater distances. Correlations among environmental predictors and PCPS axes are highlighted by black vectors. Correlations among species richness and phylogenetic diversity (PSV) and PCPS axes are highlighted by gray vectors. Black cross is the score of Boidae family (*Corallus hortulanus*), blue crosses are scores of Viperidae family (*Bothrops *species), red cross is the score of Elapidae family (*Micrurus corallinus*), green crosses are scores of Dipsadidae family, and yellow crosses are scores of Colubridae family. All species are listed in the snake phylogeny in the left‐hand side (modified from Tonini et al., 2016; see Materials and Methods), with corresponding crossing colors in ordination

The ordination of the coastal islands and snake species along the PCPS 1 and PCPS 3 axes (Figure 3) shows that species belonging to Boidae (*Corallus hortulanus*) and Colubridae families were associated with nearer and larger islands. The Elapidae family (*Micrurus corallinus*) was broadly distributed in coastal islands and does not show any significant association with the predictors. The Dipsadidae family presents a positive influence on species richness and phylogenetic diversity, whereas the Viperidae family species occur on small and more remote islands (Figure 3). Species richness, phylogenetic diversity (PSV index), and PCPS vectors did not present spatial autocorrelation (see Supporting information Figure S1).

## 4 DISCUSSION

The positive relationship between area and snake species richness follows Island Biogeography Theory predictions and matches our previous expectations, but we observed an idiosyncratic relationship of area and distance from the mainland for each diversity metrics analyzed. Phylogenetic diversity increases with distance from the mainland, independently of the decreasing richness, with regard to the phylogenetic structure distribution. More recent clades were clustered in the larger and nearer islands, whereas some older clades presented widespread distribution or defined greater phylogenetic diversity in more remote and smaller islands. Therefore, allopatric speciation events related to the occurrence of threatened endemic vipers in the Atlantic Forest island system (Barbo et al., 2016, 2012) were an important factor in these differences among richness and phylogenetic diversity.

The species‐area effect could be understood as a complementary view of habitat diversity as noted by Hortal, Triantis, Meiri, Thébault, and Sfenthourakis (2009) for several animal groups, including vertebrates and invertebrates. These authors suggest that the size of the island environments leads to a monotonical increase in the available niche dimensions, and increasing habitat diversity should be related to an increase in species richness. Therefore, the maintenance of sink populations is also associated with the available species pool and, consequently, to habitat diversity (Hortal et al., 2009). We reinforce the viewpoint that distance among assemblages and vegetation could be among the most important factors determining reptile species composition in insular environments (Guerrero, Vargas, & Real, 2005). Thus, species richness would be related to potential colonization, which might depend on area and insularity (Parent, 2012).

The relictual Atlantic Forest on islands conserves some lowland species from the southeastern coast and snakes of ombrophilous dense vegetation of Serra do Mar range (Centeno et al., 2008). Therefore, Cicchi et al. (2007) notes the fragility of these insular environments, where more than half of the snake species prey on amphibians, highlighting the importance of Forest conservation. Snake population survival and, consequently, the assemblage composition of the islands tend to be affected by distinct mechanisms such as resource availability (primary productivity and prey availability), ecological conditions, habitat selection, and environmental heterogeneity (Holt, 1993).

Our results agree in part with those of Pyron and Burbrink (2014), who, in their evaluation of island snakes on a global scale, found that phylogenetic diversity was related to isolation but not area. Herein, we found that area presented a negative relationship with phylogenetic diversity, whereas distance was positively associated to phylogenetic diversity variation, with greater weight, contradicting Island Biogeography Theory in terms of evolutionary diversity. Pyron and Burbrink (2014) also used a richness‐independent phylogenetic diversity metric (PSV) but did not explore how the different lineages were associated with the predictors of Island Biogeography Theory. Even with a greater number of species, the relatedness of species in the phylogeny, including more recent clades, corroborated the decrease in the phylogenetic diversity with increasing island area. In other words, larger islands presented phylogenetic clustered composition (Webb, Ackerly, McPeek, & Donoghue, 2002), which could indicate environmental filters defining species colonization of some specific traits (Graham, Parra, Rahbek, & McGuire, 2009; Mouquet et al., 2012; Webb et al., 2002).

The phylogenetic clustered composition in larger islands should indicate more niche similarity among species and, consequently, the possibility of competitive exclusion effects (Losos, 1996; Pausas & Verdú, 2010; Webb et al., 2002). However, the great fasting capability, annual seasonality of feeding resources, and variation in niche dimensions among species of snakes could prevent competition on populations and assemblages in this group (Vitt, 1987). Therefore, we do not consider niche similarity as a factor generating competitive exclusion of closely related species, although food and substrate requirements might limit the distribution of taxa in these assemblages. However, the dispersion of individuals from the mainland to the islands or among insular populations could reduce the extinction rates, given the proximity of this island system to the mainland (Brown & Kodric‐Brown, 1977; MacArthur & Wilson, 1963).

Most species within the coastal islands are a subsample of the mainland species pool, a situation that highlights the colonization effect as a very important driver of snake community assembly in island systems (Burbrink, McKelvy, Pyron, & Myers, 2015). However, in our results, the increase on phylogenetic diversity was related to the occurrence of endemic species of Viperidae, which led to overdispersed assemblages in smaller and more remote islands (Figure 3), independent of the species richness reduction. Differently from Burbrink et al. (2015), our results suggest the relevance of allopatric speciation to generate higher phylogenetic diversity and overdispersed assemblages in islands due to endemic *Bothrops* species on more remote islands. The maintenance of the basal clades (Viperidae) in this island system might maintain relatively longer branches among species on smaller and more remote islands, due to the occurrence of *Bothrops insularis*, *B. alcatraz,* and *B. otavioi*, on the “Queimada Grande,” “Alcatrazes,” and “Vitória” islands, respectively. These endemic species are very important components of the phylogenetic diversity and fauna conservation of the southeastern Atlantic Forest islands.

Our results suggest that principal coordinates of phylogenetic structure provide a new interpretation of the environmental factors influencing phylogenetic lineages (Duarte, 2011; Duarte et al., 2012). The “phylogeny‐weighted species composition” provides a way for us to indicate the relationships of each clade and environmental predictors as highlighted by Duarte (2011). This author shows that species scores on PCPS ordination demonstrate the phylogenetic composition throughout the environmental gradient, while also indicating the clade distributions across environmental predictors. Moreover, we provide an additional view of the disparities of diversity metrics, including species richness and phylogenetic diversity, and how snakes respond to main predictors of the classical Island Biogeography Theory.

Island area showed a greater influence on Colubridae and the single Boidae species present. These species use arboreal substrates that, in turn, depend on the availability of forested habitat. The most diversified clade, Dipsadidae, includes terrestrial, arboreal, and aquatic snakes and was also influenced by island area and mainland proximity, which would be related to habitat diversity and heterogeneity (Hortal et al., 2009). Association with particular habitats could drive the snake composition on islands (Burbrink et al., 2015). Therefore, area and distance from the mainland significantly influences snake species richness regarding to the Colubridae and Dipsadidae clades. However, decreasing area and increasing the distance from the mainland boosted the Viperidae clade, causing phylogenetic diversity maintenance, while also providing evidence of allopatric speciation in these coastal islands.

Snakes typically display high degrees of specialization in resource use (Greene, 1997), and the extinction rate of island reptiles can be related to natural history traits, including habitat specialization (Foufopoulos & Ives, 1999). Therefore, the plasticity in natural history traits and habitat use could generate a trend for greater abundances of birds in the Canary Archipelago as well as to more successful survival on islands (Carrascal, Seoane, Palomino, & Polo, 2008). Such a trend could also explain the greater occurrence of *Bothrops *species in smaller and more remote islands. *Bothrops alcatraz*, for instance, can prey mostly on centipedes and lizards, whereas *B. insularis *presents an increased venom efficiency on birds, the preferred prey of larger individuals (Martins, Araújo, Sawaya, & Nunes, 2001; Martins, Marques, & Sazima, 2002).

We corroborate the suggestion that the Brazilian southeastern islands maintain relictual snake populations of lowland species of Serra do Mar range (Centeno et al., 2008), which could be due to ecological plasticity and/or colonization success. We concluded that an island's area is more related to species richness, whereas distance from the mainland drives phylogenetic diversity on Atlantic Forest coastal islands. We empirically emphasize the importance of isolation to the evolutionary processes in driving phylogenetic diversity and lineage composition on island systems. The nearer and greater islands are very important to the maintenance of snake species richness, but we emphasize the need to conserve endemic species from isolated areas in the Atlantic Forest island system to preserve the snake phylogenetic diversity of this megadiverse domain.

## CONFLICT OF INTEREST

None Declared.

## AUTHOR CONTRIBUTION

LSOM planned the study. JTMP and LSD coordinated the study. JTMP, LSOM, LBC and TALO analyzed the data. All authors discussed the results and wrote the manuscript.

## Data Availability

The entire database has been compiled from the scientific literature, properly cited in the text, and presented in the Supporting information.
