# Supplementary material for: Area and distance from mainland affect in different ways richness and phylogenetic diversity of snakes in Atlantic Forest coastal islands
Source: Ecol Evol. 2019 Mar 14;9(7):3909–17. doi: 10.1002/ece3.5019 (PMC6468059; doi:10.1002/ece3.5019)
Supplement: Supplementary file 1 [file ECE3-9-3909-s001.doc]

**Area and distance from mainland affect in different ways richness and phylogenetic diversity of snakes in Atlantic Forest coastal islands**

José Thales da Motta Portillo^1^, Lilian Sayuri Ouchi-Melo^2^, Lucas Batista Crivellari^3^, Thiago Alves Lopes de Oliveira^4^, Ricardo J. Sawaya^5^, Leandro da Silva Duarte^4^

1- Instituto de Biociências, Letras e Ciências Exatas, Universidade Estadual Paulista “Júlio de Mesquita Filho”, Rua Cristóvão Colombo, 2265, São José do Rio Preto, São Paulo, 15054-000, Brazil.

2- Department of Biology, City College of New York, City University of New York, New York, NY, 10031, USA.

3- Departamento de Zoologia, Universidade Federal do Paraná, Rua XV de Novembro, 1299, Paraná, 80060-000, Brazil.

4- Laboratório de Ecologia Filogenética e Funcional, Depto de Ecologia, Universidade Federal do Rio Grande do Sul, Avenida Bento Gonçalves, 9500, Porto Alegre, Rio Grande do Sul, 91501-970, Brazil.

5- Centro de Ciências Naturais e Humanas, Universidade Federal do ABC, Rua Arcturus, 03, São Bernardo do Campo, São Paulo, 09606-070, Brazil.

**SUPPLEMENTARY INFORMATION**

Spatial autocorrelation is a common trend in biological data which could influence both the results and the interpretation of statistical analyses. We used Moran’s I correlograms to verify if response data (species richness, phylogenetic diversity, PCPS1, PCPS3 and PCPS4) present spatial autocorrelation trends, in order to provide a more robust interpretation of results. We did not find any spatial autocorrelation in response data (see Figure S1 below). The absence of spatial autocorrelation means that the distribution of response data values is not influenced by the distance among islands. This means that the effects of distance from the mainland and island area in response variables are not influenced by the distance among islands.


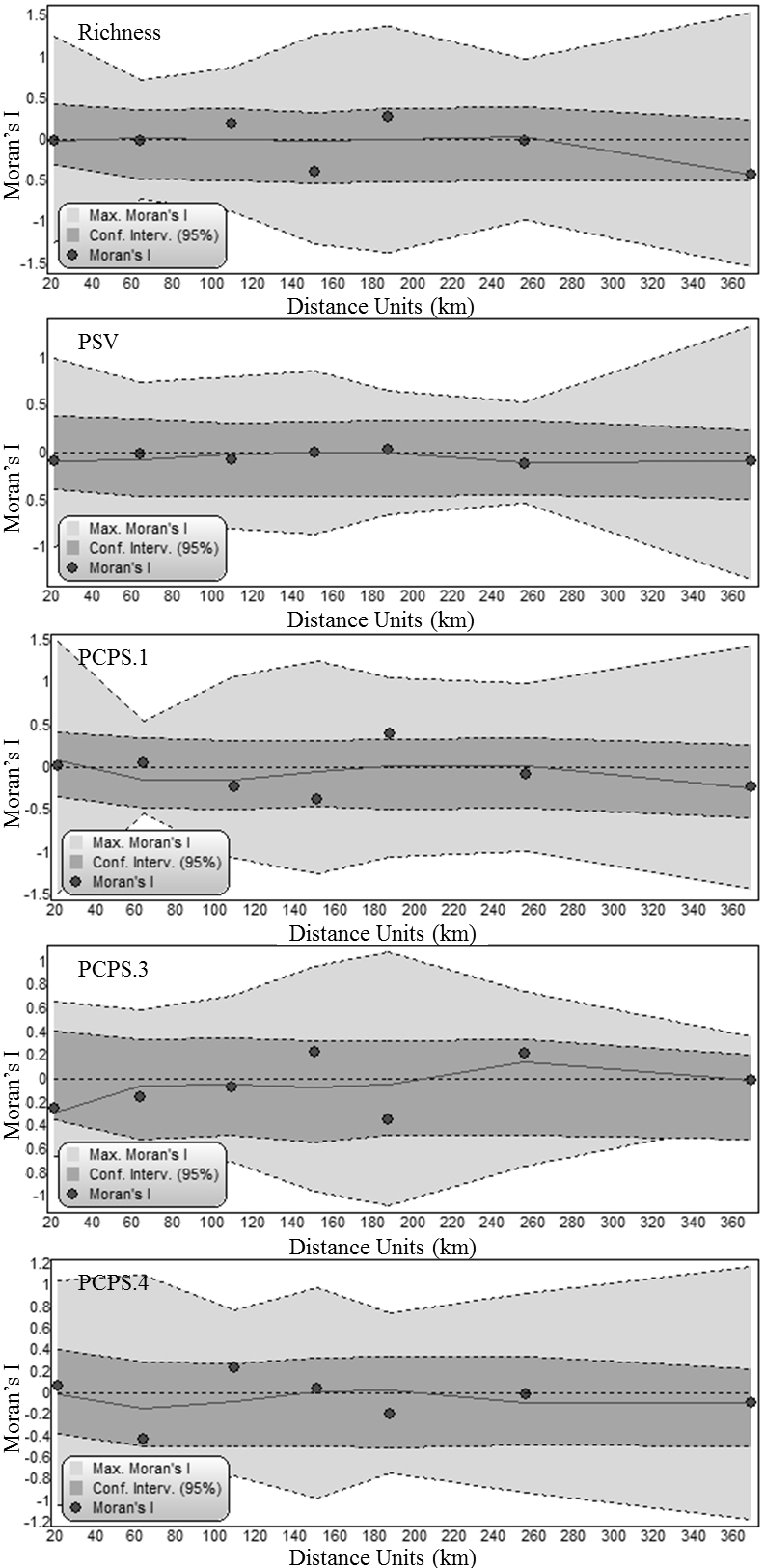


**Figure S1:** Moran’s I Correlograms for species richness, phylogenetic diversity (PSV index; see methods), and phylogenetic composition regarding PCPS vectors of snakes from coastal islands in Atlantic Forest of southeastern Brazil. Values above zero represent positive autocorrelation and values below zero represent negative autocorrelation. Values inside the envelope of 95% confidence interval (darker shade) are non-significant.
