# Supplementary material for: Area and distance from mainland affect in different ways richness and phylogenetic diversity of snakes in Atlantic Forest coastal islands
Source: Ecol Evol. 2019 Mar 14;9(7):3909–17. doi: 10.1002/ece3.5019 (PMC6468059; doi:10.1002/ece3.5019)
Supplement: Supplementary file 2 [file ECE3-9-3909-s002.doc]

**Area and distance from mainland affect in different ways richness and phylogenetic diversity of snakes in Atlantic Forest coastal islands**

José Thales da Motta Portillo^1^, Lilian Sayuri Ouchi-Melo^2^, Lucas Batista Crivellari^3^, Thiago Alves Lopes de Oliveira^4^, Ricardo J. Sawaya^5^, Leandro da Silva Duarte^4^

1- Instituto de Biociências, Letras e Ciências Exatas, Universidade Estadual Paulista “Júlio de Mesquita Filho”, Rua Cristóvão Colombo, 2265, São José do Rio Preto, São Paulo, 15054-000, Brazil.

2- Department of Biology, City College of New York, City University of New York, New York, NY, 10031, USA.

3- Departamento de Zoologia, Universidade Federal do Paraná, Rua XV de Novembro, 1299, Paraná, 80060-000, Brazil.

4- Laboratório de Ecologia Filogenética e Funcional, Depto de Ecologia, Universidade Federal do Rio Grande do Sul, Avenida Bento Gonçalves, 9500, Porto Alegre, Rio Grande do Sul, 91501-970, Brazil.

5- Centro de Ciências Naturais e Humanas, Universidade Federal do ABC, Rua Arcturus, 03, São Bernardo do Campo, São Paulo, 09606-070, Brazil.

**Appendix S1:** Occurrence of snake species in the coastal islands of the state of São Paulo, southeastern Brazil. The islands are numbered in alphabetical order: **1** = Alcatrazes, **2** = Anchieta, **3** = Barnabé, **4** = Bom Abrigo, **5** = Búzios, **6** = Cananeia, **7** = Cardoso, **8** = Comprida, **9** = Couves, **10** = Mar Virado, **11** = Porchat, **12** = Porcos, **13** = Queimada Grande, **14** = Santo Amaro, **15** = São Sebastião, **16** = São Vicente, and **17** = Vitória.

| **Family/species** | **1** | | **2** | **3** | **4** | **5** | **6** | **7** | **8** | **9** | **10** | **11** | **12** | **13** | **14** | **15** | **16** | **17** |
| --- | --- | --- | --- | --- | --- | --- | --- | --- | --- | --- | --- | --- | --- | --- | --- | --- | --- | --- |
| **Boidae** |  | |  |  |  |  |  |  |  |  |  |  |  |  |  |  |  |  |
| *Corallus hortulanus* | 0 | | 0 | 0 | 0 | 0 | 0 | 0 | 0 | 0 | 0 | 0 | 0 | 0 | 1 | 1 | 0 | 0 |
| **Colubridae** |  | |  |  |  |  |  |  |  |  |  |  |  |  |  |  |  |  |
| *Chironius bicarinatus* | 0 | | 1 | 0 | 0 | 1 | 1 | 1 | 1 | 0 | 1 | 1 | 0 | 0 | 1 | 1 | 1 | 0 |
| *Chironius exoletus* | 0 | | 0 | 0 | 0 | 0 | 1 | 1 | 1 | 0 | 1 | 1 | 0 | 0 | 1 | 0 | 1 | 0 |
| *Chironius foveatus* | 0 | | 0 | 0 | 0 | 0 | 0 | 0 | 0 | 0 | 0 | 0 | 0 | 0 | 0 | 1 | 0 | 0 |
| *Chironius fuscus* | 0 | | 0 | 0 | 0 | 0 | 0 | 1 | 0 | 0 | 0 | 1 | 0 | 0 | 1 | 0 | 1 | 0 |
| *Chironius laevicollis* | 0 | | 0 | 0 | 0 | 0 | 0 | 1 | 1 | 0 | 0 | 0 | 0 | 0 | 0 | 1 | 1 | 0 |
| *Chironius multiventris* | 0 | | 0 | 0 | 0 | 0 | 1 | 1 | 0 | 0 | 0 | 0 | 0 | 0 | 1 | 1 | 1 | 0 |
| **Dipsadidae** |  | |  |  |  |  |  |  |  |  |  |  |  |  |  |  |  |  |
| *Clelia plumbea* | 0 | | 0 | 0 | 0 | 0 | 0 | 1 | 0 | 0 | 0 | 0 | 0 | 0 | 0 | 1 | 1 | 0 |
| *Dipsas albifrons* | 1 | | 0 | 0 | 0 | 0 | 0 | 0 | 0 | 0 | 0 | 0 | 0 | 1 | 0 | 1 | 0 | 0 |
| *Dipsas alternans* | 0 | | 0 | 0 | 0 | 0 | 0 | 0 | 0 | 0 | 0 | 0 | 0 | 0 | 0 | 1 | 0 | 0 |
| *Dipsas petersi* | 0 | | 0 | 0 | 0 | 0 | 0 | 1 | 0 | 0 | 0 | 0 | 0 | 0 | 1 | 1 | 1 | 0 |
| *Dipsas neivai* | 0 | | 0 | 0 | 0 | 0 | 1 | 0 | 0 | 0 | 0 | 0 | 0 | 0 | 0 | 1 | 0 | 0 |
| *Taeniophallus affinis* | 0 | | 0 | 0 | 0 | 0 | 0 | 1 | 0 | 0 | 0 | 0 | 0 | 0 | 0 | 0 | 0 | 0 |
| *Echinanthera bilineata* | 0 | | 0 | 0 | 0 | 0 | 0 | 1 | 0 | 0 | 0 | 0 | 0 | 0 | 0 | 0 | 0 | 0 |
| *Echinanthera cephalostriata* | | 0 | 0 | 0 | 0 | 0 | 0 | 1 | 0 | 0 | 0 | 0 | 0 | 0 | 1 | 1 | 1 | 0 |
| *Echinanthera melanostigma* | 0 | | 0 | 0 | 0 | 0 | 0 | 0 | 0 | 0 | 0 | 0 | 0 | 0 | 1 | 0 | 1 | 0 |
| *Echinanthera undulata* | 0 | | 0 | 0 | 0 | 0 | 0 | 0 | 1 | 0 | 0 | 0 | 0 | 0 | 0 | 0 | 1 | 0 |
| *Erythrolamprus aesculapii* | 0 | | 0 | 1 | 0 | 0 | 1 | 0 | 0 | 0 | 0 | 0 | 0 | 0 | 0 | 1 | 1 | 0 |
| *Erythrolamprus miliaris* | 0 | | 1 | 0 | 1 | 0 | 1 | 1 | 1 | 1 | 0 | 1 | 0 | 0 | 1 | 1 | 1 | 1 |
| *Erythrolamprus poecilogyrus* | 0 | | 0 | 0 | 0 | 0 | 0 | 1 | 0 | 0 | 0 | 0 | 0 | 0 | 1 | 0 | 1 | 0 |
| *Helicops carinicaudus* | 0 | | 0 | 0 | 0 | 0 | 1 | 1 | 1 | 0 | 0 | 1 | 0 | 0 | 1 | 0 | 1 | 0 |
| *Leptodeira annulata* | 0 | | 0 | 0 | 0 | 0 | 0 | 1 | 0 | 0 | 0 | 0 | 0 | 0 | 0 | 0 | 0 | 0 |
| *Imantodes cenchoa* | 0 | | 0 | 0 | 0 | 0 | 0 | 1 | 0 | 0 | 0 | 0 | 0 | 0 | 0 | 0 | 0 | 0 |
| *Oxyrhopus clathratus* | 0 | | 0 | 0 | 0 | 0 | 1 | 1 | 0 | 0 | 0 | 0 | 0 | 0 | 0 | 1 | 1 | 0 |
| *Sibynomorphus neuwiedi* | 0 | | 0 | 0 | 0 | 0 | 1 | 1 | 0 | 0 | 0 | 0 | 0 | 0 | 1 | 1 | 1 | 0 |
| *Siphlophis pulcher* | 1 | | 0 | 0 | 0 | 1 | 1 | 0 | 0 | 0 | 0 | 0 | 0 | 0 | 1 | 1 | 1 | 0 |
| *Sordellina punctata* | 0 | | 0 | 0 | 0 | 0 | 0 | 0 | 1 | 0 | 0 | 0 | 0 | 0 | 0 | 0 | 0 | 0 |
| *Spilotes pullatus* | 0 | | 1 | 0 | 0 | 0 | 1 | 1 | 1 | 0 | 0 | 1 | 0 | 0 | 1 | 1 | 1 | 0 |
| *Thamnodynastes nattereri* | 0 | | 0 | 0 | 0 | 0 | 0 | 0 | 1 | 0 | 0 | 0 | 0 | 0 | 0 | 1 | 0 | 0 |
| *Tomodon dorsatus* | 0 | | 0 | 0 | 0 | 0 | 1 | 1 | 0 | 0 | 0 | 1 | 0 | 0 | 0 | 0 | 1 | 0 |
| *Tropidodryas serra* | 0 | | 0 | 0 | 0 | 0 | 0 | 1 | 0 | 0 | 0 | 0 | 0 | 0 | 1 | 0 | 0 | 0 |
| *Tropidodryas striaticeps* | 0 | | 0 | 0 | 0 | 0 | 0 | 1 | 0 | 0 | 0 | 0 | 0 | 0 | 1 | 0 | 0 | 0 |
| *Xenodon neuwiedii* | 0 | | 0 | 0 | 0 | 0 | 1 | 1 | 0 | 0 | 0 | 0 | 0 | 0 | 1 | 1 | 0 | 0 |
| *Xenodon merremii* | 0 | | 0 | 0 | 0 | 0 | 0 | 0 | 0 | 0 | 0 | 0 | 0 | 0 | 1 | 0 | 0 | 0 |
| **Elapidae** |  | |  |  |  |  |  |  |  |  |  |  |  |  |  |  |  |  |
| *Micrurus corallinus* | 1 | | 1 | 1 | 0 | 1 | 1 | 1 | 1 | 0 | 0 | 1 | 0 | 0 | 1 | 1 | 1 | 1 |
| **Viperidae** |  | |  |  |  |  |  |  |  |  |  |  |  |  |  |  |  |  |
| *Bothrops alcatraz* | 1 | | 0 | 0 | 0 | 0 | 0 | 0 | 0 | 0 | 0 | 0 | 0 | 0 | 0 | 0 | 0 | 0 |
| *Bothrops insularis* | 0 | | 0 | 0 | 0 | 0 | 0 | 0 | 0 | 0 | 0 | 0 | 0 | 1 | 0 | 0 | 0 | 0 |
| *Bothrops jararaca* | 0 | | 1 | 0 | 0 | 1 | 1 | 1 | 1 | 0 | 1 | 1 | 1 | 0 | 1 | 1 | 1 | 0 |
| *Bothrops jararacussu* | 0 | | 1 | 0 | 1 | 0 | 1 | 1 | 1 | 0 | 0 | 1 | 0 | 0 | 1 | 1 | 1 | 1 |
| *Bothrops otavioi* | 0 | | 0 | 0 | 0 | 0 | 0 | 0 | 0 | 0 | 0 | 0 | 0 | 0 | 0 | 0 | 0 | 1 |
